# Insights into adaptive evolution of plastomes in *Stipa* L. (Poaceae)

**DOI:** 10.1186/s12870-022-03923-z

**Published:** 2022-11-14

**Authors:** Katarzyna Krawczyk, Kamil Myszczyński, Marcin Nobis, Jakub Sawicki

**Affiliations:** 1 Department of Botany and Nature Protection, Faculty of Biology and Biotechnology, University of Warmia and Mazury in Olsztyn, Plac Łódzki 1, 10-727 Olsztyn, Poland; 2 Laboratory of Translational Oncology, Intercollegiate Faculty of Biotechnology, University of Gdańsk and Medical University of Gdańsk, Dębinki 1, 80-211, Gdańsk, Poland; 3 Institute of Botany, Faculty of Biology, Jagiellonian University, Gronostajowa 3, 30-387 Kraków, Poland

**Keywords:** cpDNA, *Stipa*, Poaceae, Positive selection, Adaptive pressure, Molecular evolution

## Abstract

**Background:**

The study presents results of research on the evolution of plastid genomes in *Stipa* L. which is a large genus of the Poaceae family, comprising species diverse in terms of geographic distribution, growing under highly variated habitat conditions. Complete plastome sequences of 43 taxa from Stipeae and Ampelodesmae tribes were analyzed for the variability of the coding regions against the background of phylogenetic relationships within the genus *Stipa*. The research hypothesis put forward in our research was that some of coding regions are affected by a selection pressure differentiated between individual phylogenetic lines of *Stipa*, potentially reducing the phylogenetic informativeness of these CDS. The study aimed to answer the question, which genes evolve in *Stipa* most rapidly and what kind of changes in the properties of encoded amino acids this entails. Another goal of this research was to find out whether individual genes are affected by positive selection and finally, whether selective pressure is uniform within the genus or does it vary between particular evolutionary lines within the genus.

**Results:**

Results of our study proved the presence of selective pressure in 11 genes: *ccsA*, *matK*, *ndhC*, *ndhF*, *ndhK*, *rbcL*, *rpoA rpoC1*, *rpoC2, rps8* and *rps11*. For the first time the effect of positive selection on the *rps8*, *rps11,* and *ndhK* genes was documented in grasses. The varied pace of evolution, different intensity and effects of selective pressure have been demonstrated between particular phylogenetic lines of the genus tested.

**Conclusions:**

Positive selection in plastid genome in *Stipa* mostly affects photosynthetic genes. The potential strongest adaptive pressure was observed in the *rbcL* gene, especially in the oldest evolutionary group comprising Central Asian high-mountain species: *S. basiplumosa*, *S. klimesii*, *S. penicillata* and *S. purpurea*, where adaptive pressure probably affected the amino acids directly related to the efficiency of CO_2_ assimilation.

**Supplementary Information:**

The online version contains supplementary material available at 10.1186/s12870-022-03923-z.

## Background

The grass family (Poaceae), which comprises ca 11,500 species representing 768 genera is the fifth largest family of flowering plants [1]. It includes species crucial from an economic point of view, let us mention here at least wheat, rice, maize, sugar cane and sorghum. More than 600 species of grasses are currently used for grazing and livestock feed [2]. Moreover, in this family there are also species of great ecosystem importance forming steppes, savannas, pampas, prairies and, finally, also lawn and ornamental species.

One of the highly represented genera in grasslands of Middle Asia is the genus *Stipa* L. (feather grass), comprising over 150 species, even if considered in a narrow taxonomic sense [3]. Its representatives are C_3_ grasses [4] distributed in warm temperate regions throughout Asia, Europe and North Africa, growing in alpine meadows, lowlands, steppes, deserts, mountains, rocks and screes [3, 5–7]. Some of *Stipa* species (*S. capillata*, *S. lipskyi*, *S. pennata*, *S. zeravshanica*) create plant communities known as feather grass steppes or feather grass grasslands, in which they are diagnostic, constant and/or dominating species [3]. Particular *Stipa* species are diverse in terms of geographic distribution. They include mountain species with a narrow range, such as *S. klimesii* growing only in the Himalayas, *S. narynica* found only in Kyrgyzstan, or *S. × heptapotamica* dispersed only in Kazakhstan. Among the feathergrasses there are also species with a very wide range, for example *S. pulcherrima*, which spans from France to the west of Siberia and Iran, or *S. capillata* which grows from central Europe to eastern Mongolia [8].

The genus *Stipa* L. according to the up to date worldwide phylogenetic classification of the Poaceae [1] belongs to the Pooideae subfamily, which together with Bambusoideae, Oryzoideae, Peulioideae, Pharoideae and Anomochloideae constitutes the BOP clade [1, 9]. *Stipa* is a typical genus of a tribe Stipeae Dumort. which scope is perceived differently by different authors. According to Schneider et al. [10] and Kellogg et al. [11] the tribe Stipeae comprises subtribe Stipiniae Griseb. and subtribe Ampelodesminae Conert. Alternatively, tribe Ampelodesmeae Tutin with only one genus *Ampelodesmos* Link is excluded from Stipeae [1, 9] as a sister group. The species typical for the genus is *S. pennata* L. [12].

*Stipa* is a large and taxonomically challenging genus, with some species highly variable in morphology [3], sometimes with very subtle diagnostic features between species [13] and in which formation of interspecies hybrids has been confirmed [14, 15]. All of this resulted in disorder in the taxonomic names that have recently been reviewed by the development of the Middle Asian *Stipa* synopsis, published along with the key for species identification [3]. In recent years, there have also been several publications devoted to reconstruct phylogenetic relationships within the genus as well as the tribe Stipeae [4, 6, 7, 16–18]. However, they were conducted using a few species and single loci, moreover, the phylogenetic signal provided by these loci was weak, and most of the relationships between species could not be resolved. In search of a more effective phylogenetic marker for this taxonomic group, a nuclear phylogenetic marker was developed which is a fragment of intergenic spacer (IGS) present in a nucleolar organizing region (NOR) [19]. In-depth study was also carried out on the variability of complete plastomes in terms of their usefulness in DNA barcoding as super-barcodes [20]. In that research, inter alia, the genetic variation of non-coding and coding regions was analyzed, and the number of observed nonsynonymous mutations in individual coding regions was counted. However, the question, whether the amino acids substituted as a result of these mutations differed significantly in properties compared to the replaced amino acids, and whether these mutations are under positive selective pressure, remained an open issue. This question is of particular importance in the light of the varied occurrence of individual species of feather grasses and the related variability of habitat conditions they have to face. Identification of genes under positive selection is also significant from the point of view of their usefulness in phylogenetic inference, as sites affected by strong positive selection generate less robust phylogenetic signal [21].

The research hypothesis put forward in our research was that some of coding regions are affected by a selection pressure differentiated between individual phylogenetic lines of *Stipa.* The aim of this study was to analyze the variability of the coding regions, in a significantly enlarged pool of plastid genomes, against the background of phylogenetic relationships within the genus *Stipa*. We aimed to answer the question, in which genes the biggest number of nonsynonymous mutations is observed, and what changes in the properties of encoded amino acids they entail. Another goal of this research was to find out whether individual genes or individual loci are under positive selection and finally, whether selective pressure varies between particular evolutionary lines or is it homogenous within the genus.

## Results

### Phylogenetic implications

**Fig. 1:**
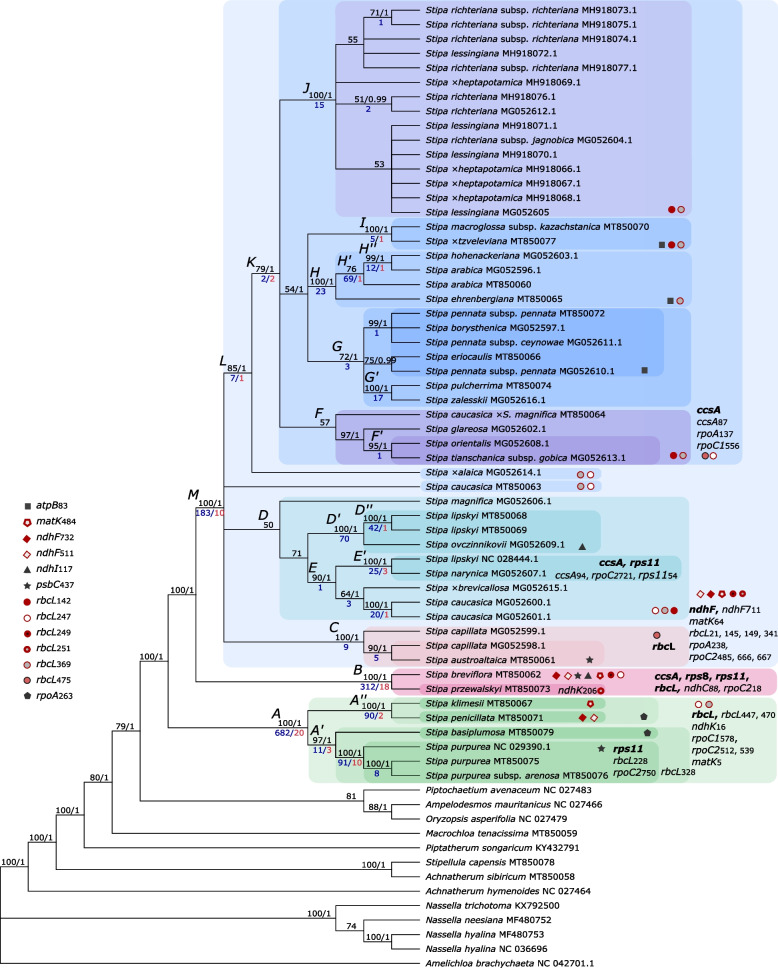
Plastome-based evolutionary analysis by Maximum likelihood method based on the GTR + Γ model of nucleotide substitution. The 50%-rule bootstrap consensus tree inferred from 1000 replicates. Bootstrap values are given in the top line before the slash. Credibility values of clades supported by Bayesian analysis are given in the top line after the slash. In the lower line the number of clade-specific mutations (in blue) and the number of nonsynonymous mutations private for particular clades (in red) are given. Clades discussed in the text and in the tables are marked with a letter. Gene names given in bold next to clades indicate clades positively selected with Clade Model. Gene names with numbers indicate sites private for clades positively selected with Site Model and/or *Z*-test. Symbols defined in the legend indicate nonsynonymous mutations observed in separate clades—for the positively selected ones the symbol is red. *clade *M* excluding *F’*

Reconstruction of phylogenetic tree for 67 sequences representing 43 analyzed species was obtained both using the Maximum Likelihood (ML) method based on GTR + Γ model and Bayesian Inference (BI). The topology of the ML bootstrap consensus tree was fully consistent with the results of BI, therefore we presented values of posterior probability (PP) next to bootstrap values (BS) in one graph (Fig. 1). All the representatives of the genus *Stipa* were grouped in one monophyletic and highly supported (BS = 100, PP = 1) clade. The remaining taxa from Stipeae tribe included in the research together with *Ampelodesmos mauritanicus* from Ampelodesmeae formed well resolved basal clades. Within *Stipa* we can distinguish two small definitely separated groups: clade *A* and *B*, both with the highest possible credibility values. The remaining samples are grouped into a highly supported (BS = 100, PP = 1) clade *M.* The analysis of plastome sequences did not allow a full resolution of all the deeper clades, thus polytomies were observed within clade *K*. Also relationships between clades: *C*, *D*, *L*, *S. magnifica* MG052606.1 and *S. caucasica* MT850063 are not completely resolved. However, external clades are in the vast majority well-supported except for clade *J* which comprises *S. lessingiana*, *S. richteriana* and their hybrid *S.* × *heptapotamica*.

### Sequence variation

**Fig. 2:**
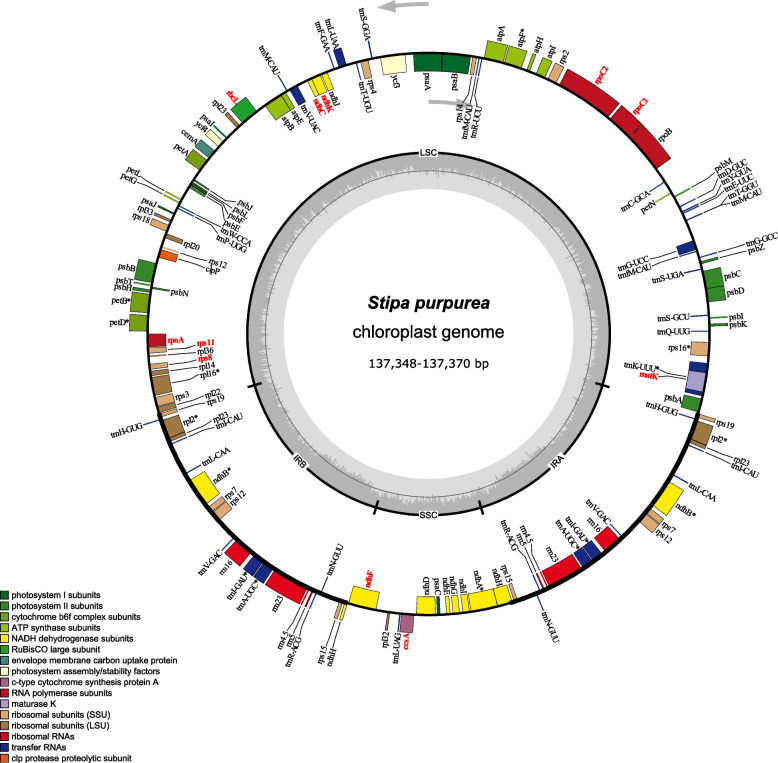
Gene map of the *Stipa purpurea* chloroplast genome. Genes positively verified for positive selection are given in red font. Dashed area in the inner circle indicates the GC content. Arrows indicate direction of transcription

In the study 67 complete plastomes were analyzed, 21 of which (18 representatives of *Stipa* and three sequences representing *Achnatherum sibiricum*, *Marochloa tenacissima* and *Stipellula capensis*) were newly sequenced and published here for the first time. For these sequences neither rearrangements of genome, nor differences in gene content and gene order were observed. De novo sequenced plastid genomes of Stipeae had a typical quadripartite structure of the large (LSC) and small single-copy (SSC) regions separated by a pair of inverted repeats (IR). Excluding the second IR region it contained 77 protein-coding genes, four rRNA genes and 32 tRNA genes. 21 genes were duplicated in IRs (Fig. 2). The annotations of *petB*, *petD*, *rpoC2 rps12*, *rpl16*, *trnM* were added relative to previously published plastomes of *Stipa* [20, 22].

The analyzed plastome sequences ranged in length from 134,281 bp to 139,946 bp. The shortest complete cpDNA sequence belonged to *Oryzopsis asperifolia*, the longest plastome was found in *Amelichloa brachychaeta*. The alignment of all the analyzed 67 plastomes was 142,757 bp long, had 7482 variable sites and was characterized by 98.9% pairwise identity. The set of sequences limited to the 54 representatives of the *Stipa* genus was 138,459 long when aligned, it was characterized by 99.7% pairwise identity and 1079 variable sites. The range of sequence length spanned from 137,202 bp in *S. klimesii* to 137,912 bp in *S. lipskyi*.

Variable nucleotides in the alignment referred to as variable sites, analyzed manually with reference to the phylogenetic tree showed high volatility in the number of mutations private for clades (Fig. 1). The most of them was observed in clade *A* and clade *B*, amounting to 682 and 312, respectively, while clade *M* was characterized by 183 private mutations. The big number of clade-private mutations did not go hand in hand with the high support values of the clade. And so, for example, clade *H’* not supported by BI, with BS = 76 had 69 private mutations. On the other hand, clades *J* and *C* having very strong support (BS = 100, PP = 1) were characterized by only 15 and nine private mutations, respectively.

Set of 77 CDS excluding the IRa region for *Stipa* was 56,283 bp long when aligned. It was variable in 761 (1.4%) sites, 371 of which came exclusively from substitutions. Nonsynonymous substitutions amounted to 103 and were distributed among 29 genes (Table 1). Beside substitutions of aa there were also instances of indels, observed within seven coding regions (Table 1). In five CDS, despite the presence of indel, the reading frame has not been changed and resulted only in addition or deletion of one (*rbcL*), two (*ccsA*, *psbT*) or seven (*rpoC2*, *rps18*) amino acids (aa). In *rpl32* and *ndhH* indels resulted in a shift of a reading frame. Thus, in the *rpl32* gene, deletion of 4 bp resulted in the substitution of one aa and elongation by four aa. The highest sequence variability was observed in the short interval of the *ndhH* gene coding from 54 aa up to 79 aa. The shortest version of *ndhH* was found in *S. breviflora*, where deletion of 47 bp resulted in its reduction by 11 aa and substitution of two aa. In the clade *H’* the deletion of the stop codon resulted in elongation of the short interval by three aa when compared to the internal *H* clade and its sister clades: *G* and *I*. In the clade *E’* duplication of 29 bp resulted in elongation of the CDS by 14 aa.

**Table 1:** Variation in genes with nonsynonymous mutations within the genus *Stipa*

CDS	Number of syn substitutions	Number of nonsyn. substitutions	Number of nonsyn. clade-specific substitutions	Isertions/ Deletions	CDS length [bp]	Number of clade-specific nonsyn. mutations per gene length	π nucleotide diversity
*atpA*	12	3	2		1524	0.00131	0.001384
*atpB*	4	3	0		1497	0.00000	0.000363
*ccsA*	7	8	6	2 aa insertion in clade *H’’*	981	0.00612	0.002172
*matK*	15	5	4		1537	0.00260	0.001504
*ndhA*	13	3	2		1089	0.00184	0.001256
*ndhC*	3	1	1		363	0.00275	0.000404
*ndhD*	15	3	1		1503	0,00,067	0.001001
*ndhF*	21	13	7		2220	0.00315	0.001784
*ndhG*	8	1	0		531	0.00000	0.002024
*ndhH*	0	1	0	duplication of 29 bp clade *E’*, del. of 3 bp in clade *H’*, del. of 47 bp in *S. breviflora*^a^	204	0.00000	0.000000
*ndhI*	3	1	1		543	0.00184	0.000583
*ndhK*	7	4	2		741	0.00270	0.001011
*petA*	1	1	1		963	0.00104	0.000221
*petB*	2	1	1		648	0.00154	0.000169
*psbA*	9	2	1		1062	0.00094	0.000724
*psbB*	8	2	1		1527	0,00,065	0.000511
*psbC*	7	2	2		1422	0.00141	0.000729
*psbT*	0	0	0	insertion of 2 aa in clade *B* and *M*	114	0.00000	0.000000
*rbcL*	12	18	17	1 aa deletion in clade *A*	3836	0.00443	0.004182
*rpl2*	5	1	0		834	0.00000	0.000089
*rpl20*	2	1	0		360	0.00000	0.000491
*rpl32*	1	1	0	deletion of 4 bp in clade *B*^a^	180	0.00000	0.000935
*rpl33*	2	1	0		201	0.00000	0.000369
*rpoA*	9	3	2		1020	0.00196	0.001414
*rpoC1*	14	5	5		2061	0.00243	0.000923
*rpoC2*	12	15	13	7 aa insertion in clade *A'*, 7 aa deletion in clade *B*	4542	0.00286	0.000772
*rps3*	8	2	1		720	0.00139	0.000836
*rps8*	1	2^b^	2^b^		411	0.00486	0.000444
*rps11*	5	4	3		432	0.00694	0.000755
*rps18*	4	3	0	7 aa in *S. penicillata*	513	0.00000	0.000815
Total	194	104	75	-	-	-	-

^a^ The effect on the coding sequence described in the text

^b^ Two substitutions resulting in the exchange of one aa

Altogether, nonsynonymous (ns) mutations were observed in 30 genes (including 29 genes with substitutions and one gene with mutation in a type of indel). All these genes are located in LSC or SSC regions beside the *rpl*2 gene which is present in IR region. The distribution of ns mutations private for specific clades is presented in Fig. 1, they are also listed in Table S2. Their biggest number is observed in clade *A*(20) and *B*(18), in clade *M* (10) and *S. purpurea* clade (10).

**Fig. 3:**
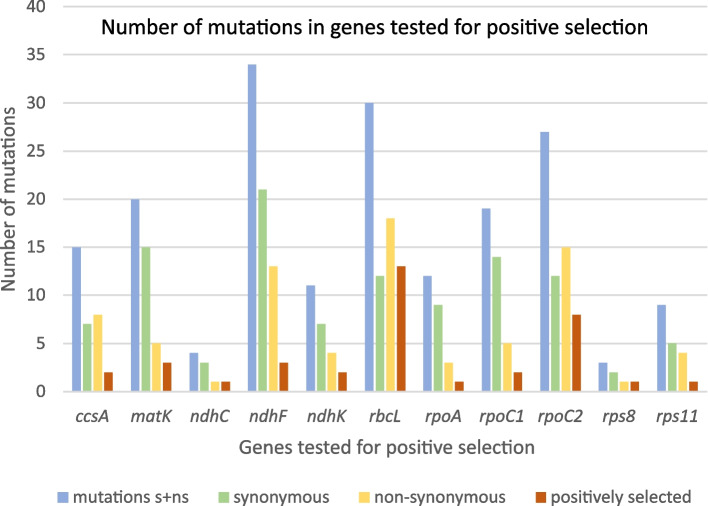
Number of mutations in genes tested for positive selection. Blue—overall number of mutations in gene, green—synonymous mutations, yellow—nonsynonymous mutations, red—sites positively selected, according to Site-Model and/or Z-Test

Apart from mutations private for single clades, some of ns mutations were also present in two or more separate and sometimes distant phylogenetic lines. The situation was observed in seven out of 30 genes where ns mutations were observed: *atpB*, *matK*, *ndhF*, *ndhI*, *psbC*, *rbcL* and *rpoA*. In the *rbcL* gene there were four sites with the same aa substitutions present in separate clades, in the *ndhF* gene—two sites and in the other genes—one such site for a CDS was observed (Fig. 3, Table S2). In *atpB* the substitution of aspartic acid with glutamic acid in site 83 is present in sister clades: *I*, *H* and *G* excluding *G’*. Similarly, in *rpoA* ns substitution appeared in closely related: *S. klimesii*, *S. penicillata* (*A*’’) and *S. basiplumosa*. In turn, the ns mutation observed in *ndhI* is shared by distant *B* and *D’* clades. In *psbC* analogical ns mutations are distributed in clade *B*, *C’* and *S. purpurea* clade.

### Positive selection

Among the 77 CDS present in plastome and 29 CDS with nonsynonymous mutations there were 21 coding regions with clade-specific mutations. 11 of them with the biggest number of clade specific ns mutations per gene length: *ccsA*, *matK*, *ndhC, ndhF*, *ndhK*, *rbcL*, *rpoA*, *rpoC1*, *rpoC2, rps8* and *rps11* were tested for the presence of positive selective pressure. The remaining ten CDS, which were not included in further analyzes, were characterized by only one or two clade-specific ns mutations, which in calculation per gene length gave very low values < 0.002. In these CDS presumed bias of positive selection on overall phylogenetic informativeness of these genes would be marginal. Secondly, testing genes with a very low proportion of ns substitutions might be prone to generating false positive results [23].

In the study four various tests for detecting positive selection: Site Model, Branch-site Model, Clade Model and the *Z*-test were applied to verify each other results and avoid making conclusions basing on uncredible results. All these methods differ from each other in assumptions (which are described in details in the Methods chapter), hence some discrepancies in the results generated by them may occur. Site-Model, which is the most commonly used in studies on selective pressure, often falsely identifies sites as positively selected, where aa substitutions are unlikely to be important in terms of functional changes. On the other hand the codon sites showing functional changes might not show a high Ω value [23]. For these reasons the results of Site-Model test were confronted with the results of *Z*-test, which is of lower power but helps selecting only aa substitutions resulting in rapid structural or biochemical changes in local properties of a peptide chain. Site-prediction methods like Site-Model and the *Z*-test take into account variable Ω in different codon sites, but do not take into account Ω variability between evolutionary lines. In the case of selective pressure acting with different strength in different evolutionary lines the Branch-site Model is useful which allows even to catch cases of relaxed positive selection. However, its application is limited to testing pressure acting on single tree branches. To study the pressure occurring in parallel in different phylogenetic lines, Clade-Model is best suited [24, 25].

Application of a Site Model in the *ccsA* gene analysis indicates the presence of a selective pressure in site 87, where in the big *K* clade threonine is replaced with arginine (Table 2). In turn, according to Branch-site analysis, selective pressure is present in site 94 in clade *E’* (LRT *P*-value = 0.514, BEB PP = 0.951), where in the *ccsA* gene serine is substituted with phenylalanine, significantly different in the property called ‘mean r.m.s. fluctuation displacement’ (PP = 0.951, confidence level = 0.95) (Table 3). The change in the aa property is qualified as category 8 which means the most radical possible change. Test statistics of the Clade Model analysis confirmed the results of the other models indicating clades *E’* and *K* as positively selected (2ΔLnL = 11.864, d.f. = 5 and *p* = 0.037) but also proved clade *B* to be under positive selective pressure (Fig. 1, Table 4).

**Table 2:** Positively selected sites on the comparison of the Site models M7 vs. M8

Gene	Length (aa)	Codon	aa positively selected	aa alternative	Branch (with positive aa)	M7 vs. M8 BEB
*ccsA*	325	87	Arg	Thr	*K*	0.996**
*matK*	512	64	Lys	Arg	*M*	0.987*
		484	His	Leu	*B*, *M*, *S. klimesii*	0.999**
*ndhF*	740	511	Asp	Asn/His	*B*, *M*, *S. penicillata*	0.985*
		711	Val	Ile	*M*	0.962*
		732	Val	Met	*B*, *M*, *S. penicillata*	0.990*
*rbcL*	475	21	Arg	Lys	*M*	0.979*
		142	Ile	Val	*D*, *F*, *I*, *J*	1.000**
		145	Ser	Ala	*M*	0.982*
		149	Asn	Glu	*M*	0.976*
		247	Val	Cys	*A*, *B*, *D*, *S. x alaica*, *S. caucasica MT850063*	1.000**
		249	Asp	Glu	*B*, *M*	0.966*
		251	Met	Ile	*M*	0.998**
		251	Met	Leu	*S. przewalskyi*	0.998**
		341	Ile	Met	*M*	0.978*
		369	Ala	Val	*A*,* M* excluding *C and G*	0.999**
*rpoA*	340	137	Val	Ala	*K*	1.000**
		238	Asn	Ser	*M*	0.979*
		265	Asn	Lys	*A* excluding *S. purpurea*	0.985*
*rpoC1*	683	556	Tyr	Asn	*K*	1.000**
*rpoC2*	1521	485	Asn	Ser	*M*	0.955*
		666	Gly	Asp	*M*	0.959*
		667	Lys	Glu	*M*	0.955*

Posterior probability calculated with the Bayes empirical Bayes method are marked according to the confidence level: *—0.95; **—0.99; ***—0.999

**Table 3:** Amino acid properties positively selected with *Z*-test in reference to localization on a phylogenetic tree

Gene	Codon	Clade	Property
*ccsA*	94	*E'*	(8*) Mean r.m.s. fluctuation displacement
*matK*	5	*A*	(8*) Alpha-helical tendencies
	484	*B*, *M*, *S. klimesii*	(7**) Hydropathy, (7*) Polarity
*ndhC*	88	*B*	(8*) Equilibrium constant (ionization of COOH)
*ndhK*	16	*A*	(6***) Power to be at the C-terminal
	206	*S. przewalskyi*	(6*) Short and medium range non-bonded energy
*rbcL*	142	*D*, *F*, *I*, *J*	(8***) Equilibrium constant (ionization of COOH)
	145	*M*	(6***) Turn tendencies
	149	*M*	(6***) Turn tendencies
	228	*S. purpurea*	(6***) Turn tendencies
	247	*A*, *B*, *D*, *S. x alaica*, *S. caucasica MT850063*	(6***) Turn tendencies
	249	*B*, *M*	(6***) Turn tendencies
	251	*M*, *S. przewalskyi*	(8***) Equilibrium constant (ionization of COOH)
	328	*A'*	(6***) Turn tendencies
	341	*M*	(8***) Equilibrium constant (ionization of COOH)
	447	*A*	(8*) Compressibility
	470	*A*	(8*) Compressibility
	475	*C*, *K*	(8***) Equilibrium constant (ionization of COOH)
*rpoC1*	578	*A*	(6***) Power to be at the C-terminal
*rpoC2*	18	*B*	(8*) Helical contact area, (6*) Molecular volume, (7*) Molecular weight, (7*) Normalized consensus hydrophobicity, (7*) Partial specific volume, (6*) Refractive index
	512	*A*	(8*) Helical contact area, (6*) Molecular volume, (7*) Molecular weight, (7*) Normalized consensus hydrophobicity, (7*) Partial specific volume, (6*) Refractive index
	539	*A*	(8**) Isoelectric point, (7*) Power to be at the C-terminal
	666	*M*	(7*) Power to be at the C-terminal
	667	*M*	(8**) Isoelectric point, (7*) Power to be at the C-terminal
	721	*E'*	(7*) Power to be at the C-terminal
	750	*S. purpurea*	(7*) Power to be at the C-terminal
*rps8*	73	*B*	(6**) Coil tendencies, (6*) Total non-bonded energy
*rps11*	54	*E'*	(6*) Thermodynamic transfer hydrohphobicity

Number in brackets 6–8 refer to categories of most radical changes an aa properties. Confidence level: *—0.95; **—0.99; ***—0.999

**Table 4:** Branches positively selected with the Clade Model. Evolutionary model compared are M2a_rel vs CmC

Gene	Foreground branches	2ΔLnL	df	*P*-value
*ccsA*	*B*, *E'*, *K*	11.864	5	0.03670
*ndhF*	*M*	7.038	3	0.02963
*rbcL*	*A*, *B*, *C*	24.285	3	0.00002
*rps8*	*B*	17,065	1	0,00,004
*rps11*	*B*, *S. purpurea*, *E'*	443.257	3	0.00000

In the *matK* gene three sites are under positive selective pressure (Fig. 3). Site Model indicated on site 64, where in clade *M* arginine is substituted with lysine. Also site 484 where leucine is replaced with histidine is positively selected in the whole clade *M*,* B* and *S. klimesii*. The substitution causes the radical change (category 7) of two aa properties: hydropathy and polarity (Fig. S2). The results of the *Z*-test also proved the presence of positive selection in codon 5. In this site glutamic acid is substituted with glycine exclusively in clade *A* which has a strong effect (category 8) on ‘alpha-helical tendencies’ (Table 3).

For the *ndhC* gene the *Z*-test was the only one with positive result. Mutation of site 88 in clade *B* had an effect on the ‘equilibrium constant’ (Table 3).

In the case of the *ndhF* gene test statistics of the Clade Model proved clades *M* to be under positive selection (2ΔLnL = 7.038, d.f. = 3 and *p* = 0.030). None of the sites was positively verified by the *Z*-test BEB analysis, however, the Site Model positively verified three of them. In site 711 the positively selected aa was present in clade *M* and in two others (511 and 732) in clade *M*, clade *B* and *S. penicillata*.

As in the case of *ndhC*, only the *Z*-test suggested the presence of a positive selection in the *ndhK* gene. In clade *A* single nucleotide polymorphism causes a significant change in the property referred to as ‘power to be at the C-terminal’ (Table 3). In the case of *S. przewalskyi* in site 206 there is one mutation present resulting in a change from tyrosine to asparagine which results in a significant modification of ‘short and medium range non-bonded energy’.

In the *rbcL* gene according to the Clade Model analysis, adaptive pressure is present in three clades: *A*, *B* and *C*. Test statistics show that 2ΔLnL = 24.285 significantly bigger than df = 3 and *P*-value = 0.00002. Site Model analysis suggested nine sites as positively selected while the *Z*-test proved 12 sites to be under destabilizing selection each of them affecting a single rapid change in aa property. Four of these positively selected mutations are exclusive for the *M* clade, another two are shared by clade *M* and clade *B* (site 249) or *S. przewalskyi* (251). The other positively selected aa are variously distributed on the phylogenetic tree (Fig. 1). Interestingly, positively selected mutations had an effect on only three properties: ‘turn tendencies’, ‘compressibility’ and ‘equilibrium constant’.

All three nonsynonymous mutations observed in *rpoA* were positively verified by test using Site Model. Two of them were clade-specific and occurred in clade *K* (site 137) and *M* (site 238) (Table 2). The third one was observed in clade *A* excluding *S. purpurea*. However, none of the other tests confirmed this result, the *Z*-test showed no rapid change in aa properties, therefore the presence of positive selection in *rpoA* remains questionable.

In the case of *rpoC1* results of various tests are divergent. The Site Model indicated on site 556 and clade *K* (aspartic acid to tyrosine) (Table 2), while the *Z*-test suggested site 578 to be under positive pressure (aspartic acid to asparagine in clade *A*), where it results in a change of encoded aa in ‘power to be at the C-terminal’ (Table 3).

The Site Model analysis proved three sites to be positively selected in *rpoC2* (Table 2), in each of these, the amino acid substitution applied to clade *M.* Selective pressure in two of them: site 666 (aspartic acid to glycine) and 667 (glutamic acid to lysine) is confirmed by the *Z*-test. The effect of the first substitution is a change in ‘the power to be at the C-terminal’ while the second one causes rapid changes in two aa properties: ‘isoelectric point’ and ‘power to be at the C-terminal’. Moreover, the *Z*-test results indicated positive destabilizing selection in five more sites, concerning clade *A* (two sites), clade *B*, clade *E’* and the *S. purpurea* clade. Substitutions in site 512 (clade *A*) and 18 (clade *B*) are especially interesting as each of them results in radical changes of six aa properties, in other sites one or two properties are modified (Table 3).

In the *rps8* gene two nonsynonymous substitutions are both present in site 73 in clade *B* changing glycine to glutamine which significantly differs in properties ‘coil tendencies’ and total non-bonded energy’ (Table 3). The result of positive selection acting on this site was also supported by Branch-site Model and Clade Model analysis (Tables 5 and 4).

**Table 5:** Genes positively verified for the presence of positive selective pressure with different statistical models

CDS	Site Model	Branch-site Model	Clade Model	*Z*-test
*ccsA*	X	X	X	X
*matK*	X			X
*ndhC*				X
*ndhF*	X		X	
*ndhK*				X
*rbcL*	X		X	X
*rpoA*	X			
*rpoC1*	X			X
*rpoC2*	X			X
*rps8*		X	X	X
*rps11*			X	X

In the *rps11* gene the Clade Model method clearly indicated the existence of selection pressure in the *E’* clade in site 54 (2ΔLnL = 443,257, d.f. = 3 and *p* = 0.000) (Table 4). The site is also the only one positively verified by BEB analysis as well as by *Z*-test. Substitution of proline with serine in this site causes radical change in ‘thermodynamic transfer hydrophobicity’. The Clade Model analysis also proved the presence of positive selection acting on clade *B* (serine to asparagine, site 25) and clade grouping *S. purpurea* representatives (arginine to lysine, site 13) (Table S2).

### Mutational hotspot regions

11 genes tested for the positive selection were also analyzed for the spatial differentiation of substitution rates in order to check if positively selected sites coincide with hotspots of sequence variation. The analysis of substitution rates within genes showed that *rpoA*, *rpoC1* and *rpoC2* genes are mutating quite evenly along their entire length and only the first 500 bp region in *rpoC1* is conservative (Fig. S1). All the variable sites have uniform, very high rates of substitution. Location of variable sites is also even within *rbcL* and *ndhF* genes, however substitution rates are highly varied between sites. High and quite uniform rates of substitution characterize the *matK* gene. Similar to *rpoC1* there is a long conservative sequence located between 192 and 578 bp.

In the *ccsA* gene both hot-spots of variation and conservative regions are present, while substitution rates between variable sites are moderately differentiated. The distribution of variable sites looks interesting in *ndhK*, as there are only seven of them, all of them belonging to the same class rate but located in two groups: at the beginning and near the end of the CDS. In the *rps11* gene there are only five variable sites, distributed more evenly than in *ndhK*, assigned to one rate class. The lowest number of substitutions equal to three is observed in *ndhC* and *rps8*.

## Discussion

Among the CDS which were tested in our study for positive selection and have been positively verified in terms of the presence of selective pressure, there are genes falling into different functional categories, described in Table 6. The first category are genes that encode for the plastid genetic apparatus. Under this category fall the *rpo* genes encoding subunits of plastid-encoded plastid polymerase PEP. This DNA-directed RNA polymerase is one of the key enzymes that maintain the semi-autonomous nature of the plastid [26, 27]. All four genes encoding PEP subunits are present in the LSC region, however only three of them: *rpoB*, *rpoC1 and rpoC2* are found in one gene cluster, together with *rps*2 [28]. Despite coding for one enzyme, functioning within the same operon, and having a similar level of nucleotide diversity, the power of selective pressure differs between the *rpoC1* and *rpoC2* gene in *Stipa*, even considering differences in length of the genes. While in the case of *rpoC1* only two sites are under positive pressure, in the *rpoC2* gene as many as eight sites are positively selected. Interestingly, the selective pressure only affects the 5’ part of the gene between 18 and 750 aa (while the entire product is over 1500 aa long), although nonsynonymous substitutions are scattered all over the CDS. The *rpoC2* gene is one of the most rapidly evolving genes in feather grasses, considering a large share of nonsynonymous substitutions, extensive indels, as well as rates of substitution which are very high and evenly distributed over the gene in comparison to other genes analyzed in the study, but also compared to substitution rates observed in *rpoC2* and *rpoC1* in *Lamium* [29]. The *rpoA* gene, which is placed in different cluster than the rest of *rpo* genes [28] undergoes positive selection only in two aa residues, however one of them is present in the same clade (*K*) as in the case of *rpoC1* (Fig. 1) and the other one, characteristic for clade *M* corresponds to *rpoC2*.

**Table 6:** Functional characteristic of genes with nonsynonymous mutations observed within the *Stipa* genus

Genes Category	Group of Genes	Name of Genes
Transcription and post-transcriptional modification	DNA-dependent RNA polymerase	***rpoA****, ****rpoC1****, ****rpoC2***
	Maturase	***matK***
Proteins for translation	Small subunit of ribosome proteins	*rps3, ****rps8****, ****rps11****, rps18*
	Large subunit of ribosome proteins	*rpl2, rpl20, rpl32, rpl33*
Light-dependent proteins of the photosynthetic light reactions	Subunits of NADH-dehydrogenase	*ndhA, ****ndhC****, ndhD, ****ndhF****, ndhG, ndhH, ndhI, ****ndhK,***
	Subunits of photosystem II	*psbA, psbB, psbC, psbT*
	Subunits of cytochrome b_6_/f complex	*petA, petB*
	Subunits of ATP synthase	*atpA, atpB*
Light-independent proteins related to photosynthetic dark reactions	Large subunit of RuBisCO	***rbcL***
	Cytochrome c biogenesis protein	***ccsA***

Genes where positive selection was detected are given in bold

Another gene coding for an enzyme from the “plastid genetic apparatus” category, verified as being positively selected, is the *matK* gene which product is referred to as maturase K, acting as splicing factor for plastid group IIA introns [30]. In previous studies it has been reported as fast evolving, with nearly equal substitution rate at all codon positions [31]. Such a pattern of substitution indicates relaxed purifying selection [32], which led some researchers to question MatK function in land plants [33]. More general role of *matK*-encoded maturase might be suggested by the research results, which confirmed the presence of purifying selection acting on *matK* in Orobanchaceae and some *Cuscuta* species [33], what may be evidence of its sustained functionality, despite the parasitic nature of this plants. Our results, on the other hand, showed the uneven substitution rate within *matK* in *Stipa* as well as the presence of selective pressure, which speaks for the unequal evolution rate between individual gene fragments, and thus for maintaining its functionality.

Within the genes involved in the translation process, positive pressure was suggested in the case of the *rps11* gene, which is present in the LSC region, in a gene cluster together with six other ribosomal protein genes and the *rpoA* gene [28]. The presence of positive selection on *rps11* observed in particular phylogenetic lines within *Stipa* is quite surprising as previous research carried out on Poaceae PACMAD clade proved purifying or neutrality selection on this gene on the whole tree [34]. Admittedly, in that study only one hypothesis was tested with Branch-site Model, assuming there was a difference in selective pressure acting on C_4_ and C_3_ species, however individual phylogenetic lines have not been tested. Thus, according to the authors' best knowledge, *Stipa* is so far the only grass genus in which the presence of positive selection on *rps11* was demonstrated.

The other genes affected by positive selective pressure in *Stipa* are involved in the photosynthesis process. Three of them: *ndhC*, *ndhF* and *ndhK* are related to light-dependent proteins of the photosynthetic light reactions. All of them code for subunits of the plastid NAD(P)H-dehydrogenase complex (Ndh1-complex) located in the thylakoid membrane [35]. The Ndh1-complex may also be associated with other pathways like chlororespiration and might play an important role in adaptation to environmental stress [33]. It has been shown that nitrogen starvation affected (up-regulated) *ndh*-gene expression indicating a putative regulating function of Ndh1 for the photosynthetic electron flow [36, 37]. The *ndhF* gene turned out to be one of the most variable plastid genes in *Stipa*, characterized by the biggest number of nucleotide substitutions of which more than a third was nonsynonymous. The presence of selective pressure in *ndhC* and *ndhF*, demonstrated in our study for particular aa residues in the whole phylogenetic tree or for its individual branches, is a result consistent with previous studies on Poaceae [34]. In turn, in other monocots studied so far, the evolution of *ndhC, ndhF* and *ndhK* seems not to be influenced by adaptive evolution [38, 39].

Within the genes coding for light-independent proteins, related to photosynthetic dark reactions, *ccsA* and *rbcL* were verified as being positively selected. The *ccsA* is a gene which encodes a protein mediating the attachment of heme to c-type cytochromes during cytochrome biogenesis [40]. The gene usually is conserved among photosynthetic plants [33], however in grasses positive selection acting on particular sites in *ccsA* have been proved [34]. Our research proved this short gene to be rapidly evolving in *Stipa*, what can be inferred from its high level of nucleotide diversity, spatially diversified substitution rate, high proportion of nonsynonymous mutations and evidence of positive selective pressure observed in distant phylogenetic lines.

The *rbcL* gene, which encodes the large subunit of the ribulose-1,5-bisphospate carboxylase/oxygenase (RuBisCO)—one of the key photosynthetic enzymes, is probably also involved in photosynthesis unrelated pathways, what can be inferred after its retention, expression and evidence for strong purifying selection in parasitic plants [41, 42]. In *Stipa* the *rbcL* gene was characterized by the biggest nucleotide diversity among CDS, advantage of nonsynonymous over synonymous mutations and the biggest number of positively selected sites. The previous studies on PACMAD grasses [34, 43] demonstrated that *rbcL* underwent strong positive selection during the C_3_-C_4_ photosynthetic transitions, particularly the 3’ end of the gene. The C-terminal part of RbcL (positions downstream from 460), in particular site 470 [44] (referred to as 471 in Piot et al. [34]), is involved in the opening/closing mechanism of the active site of the RuBisCO [44], therefore aa substitutions in this protein region affect RuBisCO catalytic activity by modulating the opening speed of the active site [44, 45]. According to Schlitter and Wildner [46] longer opening time of the active site prefers O_2_ binding, while shorter availability of active site allows for higher CO_2_ specificity and is considered as an optimization of enzyme activity for C_4_ photosynthesis. In PACMAD grasses ten sites have evolved under positive selection in C_4_ species, moreover in C_4_ species a deletion of one aa (site 477) in the 3’ end of *rbcL* was observed. Five of these sites (142, 145, 328, 470 and 475) have also been positively selected in the *rbcL* gene in *Stipa*, however only in the case of site 328 and 470 the same type of aa substitution was selected. Selective pressure on site 328 is observed in clade *A’*, while on site 470—in clade *A*, same as the deletion of the 477 aa. These results suggest that in *Stipa*, in the phylogenetic line leading to clade *A*, positive selection fixes mutations that are directly related to the CO_2_ binding efficiency.

The presence of selective pressure in *Stipa* plastomes is evidence of adaptive evolution, although indication of specific phenotype adaptations cannot be specified at this level of studies. Only in the case of some mutations observed in RuBisCO we can predict that they are related to an increase in the efficiency of CO_2_ binding. This potential adaptation observed in *Stipa* refers to four high-mountain species (*S. basiplumosa*, *S. klimesii*, *S. penicillata and S. purpurea*) of Central Asian range and is probably related to living in rarefied air conditions as these species are reaching an altitude of up to 4500–4700 m above sea level [3].

Strong pressure on photosynthetic genes is also observed in the remaining species of feather grasses, although it is not clear what adaptations they may affect. In the case of large clade *M*, which comprises species with a diverse, often wide geographic range, different habitat preferences and vertical distribution, it can be assumed that positive mutations are inherited from a common ancestor and should be explained rather by its history and habitat conditions.

The analysis of positively selected sites, including those entailing rapid changes in aa properties, showed that the majority of these mutations are characteristic for basal clade *A*, clade *B* or they distinguish clade *M* from *A* and *B*. In these clades numerous cases of selective pressure are associated with a large number of clade-private mutations and they could be taken as its derivative. The large number of clade-private mutations observed in basal clades and in *M* group allows us to infer about the former separation of these phylogenetic lines. In turn, definitely lower numbers of clade-private mutations observed within *M* clade might suggest numerous phenomena of gene flow between plastid genomes at various stages of the development of this evolutionary line.

Although the reconstruction of phylogenetic interconnections was not the mainstream of our study, the obtained results in our opinion merit some comment. Both phylogenetic trees reconstructed in our study with two different methods—Maximum Likelihood based on GTR + Γ model of nucleotide substitution and Bayesian Inference with partitions separately modeled—were of highly convergent topology. The reason for this convergence may be due to the overall low evolutionary rate of analyzed sequences. Despite the conserved sequences of *Stipa* plastomes, observed variation provided useful phylogenetic information at the inter- and infrageneric level. Compared to the previous study, where phylogenetic implications were based on complete plastomes of 19 taxa representing the genus of *Stipa* [20], this study with a much larger set of species widened the picture of plastome-based phylogeny and confirmed phylogenetic relationships resolved previously. Similarly, referring our results to the previous studies [6, 7, 18], where variability of single or multiple loci from plastid genome or nuclear ITS was used in phylogenetic inference, we observe increased resolution or confirmation of previously resolved relationships within *Stipa* representatives. Compared to the results based on nuclear IGS [19], which had 17 species in common with present study, the plastome analysis did not confirm the tree topology calculated on the basis of IGS only in the case of *S. arabica*. The results of the nuclear sequence analysis suggested an earlier separation of the lineage leading to *S. arabica* and their closer relationship with *S. przewalskyi*, while the plastome analysis placed it on the phylogenetic tree in a group which is sister to *S. pennata* and its close relatives.

If we look at the intergeneric relations in Stipeae, we see that in general they confirm earlier reports, both those based on the analysis of complete plastomes [47] and the multi-locus plastid non-coding regions [6]. Differences in a phylogeny reconstruction concern the position of *Macrochloa tenacissima*, which was basal for Stipeae in Romaschenko et al. [6] while our results show that the phylogenetic line leading to this species separated far later and is more closely related to *Stipa* than has been suggested before. As to the taxonomic position of *Ampelodesmos*, our plastome-based phylogenetic analysis strongly points to the lack of its separateness from Stipeae tribe, thus it does not prove the need to create a separate tribe Ampelodesmeae.

## Conclusions

We present results of thorough study on evolution of plastomes in the *Stipa* genus represented by 35 taxa from 31 species. We have analyzed variability of all the protein-coding genes and tested 11 of them, characterized by the biggest number of clade-specific nonsynonymous mutations per gene length, for the presence of adaptive evolution events, revealed as positive selective pressure affecting particular sites in the gene or particular branches of the phylogenetic tree. Positively selected nonsynonymous substitutions were checked for entailing rapid changes in properties of amino acids. We have also plotted substitution rates and the likelihood of adaptive pressure occurrence in particular regions of the tested genes to visualize the spatial differences in the rate of their evolution.

Results of our study indicate the presence of selective pressure in various evolutionary lines of feather grasses, which mainly concerns genes related to the photosynthesis process and DNA-dependent RNA polymerase genes. For the first time in Poaceae, the effect of positive selection on the *rps8*, *rps11* and *ndhK* genes was documented.

The results of our study suggest that the *rbcL* gene is under the strongest adaptive pressure, especially in the oldest evolutionary group, including high-mountain species, where adaptive pressure affects the amino acids directly related to the efficiency of CO_2_ assimilation. Positive selection in *rbcL* should be taken into account when this gene is used for phylogenetic reconstructions.

## Methods

### Taxon sampling and data acquisition

In the study 67 complete plastome sequences were used from the 43 species belonging to the tribe of Stipeae and Ampelodesmae, including 54 sequences representing 34 taxa from the *Stipa* genus. We have authored 56 sequences while 11 were downloaded from the GenBank (Table S1). The 21 plastome sequences were developed solely for this article and have not been previously published (GenBank number of newly sequenced plastomes are given in Table S1). Some of the specimens used in the study (36 samples) were collected in wild in the years 2011–2019 and deposited in Herbarium of Jagiellonian University in Kraków, Poland, the others were taken from herbaria (Table S1). Plant material collection complied with local and national regulations. Sampling was conducted exclusively on public land. For collection of species protected in Poland official consent was given by Regional Directorates for Environmental Protection in Bydgoszcz and Gorzów Wielkopolski. (Decisions No. WPN.6400.16.2013.JC.1, WPN.6400.26.2015.JC, WPN-I-6205.25.2015.AI, WPN-I.6400.61.2014.AT.). Collection of plant material conducted in Czech Republic, Kazakhstan, Kyrgyzstan and Tajikistan did not require any permits, as the collected plant species are not protected by law in these countries. Due to the large number of collected specimens, detailed data on the source of the material with a description of the sampling site are given in Table S1. Plant names were accepted after Nobis et al. [3].

### Plastid genome sequencing and assembly

The libraries developed for the newly published genomes were prepared using KAPA Hyper PLUS (Roche) and sequenced using HiSeq4000 sequencer (Illumina) by Macrogen Inc. (Korea). To confirm the structure of IRs the long-read library was constructed for *S. macroglossa* subsp. *kazachstanica* using Ligation Sequencing Kit SQK-LSK109 (Oxford Nanopore Technologies) and NEBNext® Companion Module for Oxford Nanopore Technologies® Ligation Sequencing (New England Biolabs) according to manufacturer’s protocol and sequenced using MinION MK1B portable device (ONT) and R.9.4 Flow Cell (ONT). The Flow Cell was prepared for sequencing with Flow Cell Priming Kit EXP-FLP002 (ONT). Sequence reads were basecalled using high-accuracy guppy basecalling on the MinKNOW platform.

The details on library preparation, validation, quantification and sequencing of previously published genomes are given in Myszczyński et al. [22], Krawczyk et al. [20], Nobis et al. [14]. Complete plastid genomes were assembled and annotated according to previously published pipelines [22, 48].

A physical map of the plastome was generated using OGDRAW 1.2 [49].

### Phylogenetic reconstruction

Phylogenetic analyses were carried out on a set of 67 complete cpDNA sequences. The sequences were aligned using progressive Mauve algorithm in Geneious Prime [50], checked manually and trimmed. Inverted Repeat A (IRa) was found in Geneious Prime with Find Repeat function and excluded from dataset. Bayesian inference (BI) and Maximum likelihood (ML) methods were applied for reconstruction of phylogenetic relationships.

Prior to Bayesian analysis, PartitionFinder2 [51] was used to determine the best partitioning schemes and corresponding nucleotide substitution models. The data-set blocks were predefined a priori based on protein coding genes (CDS) and intergenic spacers as well as for first, second and third position for each of CDS. The Bayesian information criterion (BIC) and the ‘greedy’ algorithm with branch lengths estimated as unlinked were used to search for the best-fit scheme. BI was conducted using MrBayes 3.26 [52], and the MCMC algorithm was run for 5,000,000 generations (sampling every 500) with four incrementally heated chains (starting from random trees). The first 10% of overall trees were discarded as burn-in, and the remaining trees were used to develop a Bayesian consensus tree.

ML analysis was conducted using MEGAX based on the GTR + Γ model of nucleotide substitution. The Bootstrap consensus tree was inferred from 1000 replicates. Initial trees for the heuristic research were obtained automatically by applying Neighbor-Join and BioNJ algorithms. A district Gamma distribution was used to model evolutionary rate differences among sites (7 categories; parameter = 0.1000).

### Detection of a positive selection pressure and patterns of substitutions

Number of variable nucleotides, synonymous and nonsynonymous mutations as well as π diversity were calculated in MEGAX. Clade-specific nonsynonymous mutations were analyzed in a set of sequences limited exclusively to representatives of the *Stipa* genus to avoid disrupting the analysis by inclusion of reversions potentially present in evolutionarily more distant species. 77 coding regions were analyzed for the presence of nonsynonymous mutations and in particular the presence of nonsynonymous substitutions present in whole clades (*i.e.* clade-specific). From the set of 21 CDS characterized by the presence of nonsynonymous clade-specific substitutions, 11 genes with the biggest number of clade-specific nonsynonymous mutations per gene length were checked for the occurrence of positive selection with EasyCodeML software [25]. Each of the 11 coding regions was separately analyzed under the Site, Branch-site and Clade Model. The Site Model was used to identify positively selected sites in a sequence alignment assuming that the substitution rate ratio (ω) is the same across branches of phylogeny but heterogeneous among sites in the alignment [24]. The Site Model tested the fit of seven codon substitution models: M0, M1a, M2a, M3, M7, M8 and M8a [25] to the sequence data and compared this fit using likelihood-ratio tests (LRT) [24]. The same set of data was analyzed with a Branch-site Model to identify signals of episodic selection occurring along specified branches [53]. Model A, which allows positive selection in a selected branch, was compared against a null model (Model A_null_) that allows neutral evolution and negative selection [54].

Clade Model that accommodates site-specific divergence in selective constraint among clades was also applied [55]. The Model C (CmC), which estimates a separate ω ratio for each of two or more clades, was compared against a null model 2a_rel (M2a_rel) in which ω is fixed among clades [56]. If LRT yielded a significant value for any of the pairwise comparisons of models, the Bayes empirical Bayes (BEB) method was then applied to identify particular amino acid residues which have potentially evolved under positive selection [25].

Detection of a positive selection pressure at the protein level was also studied using software TreeSAAP v. 3.2 [57]. The influence of nonsynonymous mutations on 31 structural and biochemical aa properties were tested for correlation with a phylogenetic ML tree. A gradient of eight categories was used to classify each property change, with lower categories indicating more conservative changes. *Z*-test was performed to determine if observed changes deviated from neutral expectations [58]. Positive destabilizing selection on amino acid property was detected when the frequency of radical changes (6, 7 or 8 category) exceeded the frequency expected by chance, as indicated by positive *Z*-scores. Testing the influence of the observed changes on the properties of the peptide chain was carried out for the 15-aa-long fragments, therefore the results should be interpreted as local changes. The impact of these changes on the properties of the whole protein would require further research, including, for example, protein modeling.

The distribution of substitution rates across sites were estimated with HyPhy [59] by assigning a rate class to each site based on the GTR model using Substitution Rates analysis according to the method described in [29].

Clade-specific mutations for individual clades were calculated according to the Jörger and Schrödl [60] approach using FASTACHAR software [61].

## Supplementary Information

**Additional file 1: Table S1. **

**Additional file 2: Table S2. **

**Additional file 3: Table S3.**

**Additional file 4: Figure S1. **Distribution of substitution rates across 11 genes as calculated in HyPhy using the GTR model of evolution.

**Additional file 5: Figure S2. **The likelihood of adaptive pressure occurrence in particular CDS. The plots show the results of the *Z*-test, carried out for the 15-codon-long fragments. The values shown in the Y-axis (Z-scores) above 2.33 (blue horizontal line) correspond to the reliability level of 99%. Values above 3.09 (red horizontal line) correspond to the reliability of 99.9%.

## Data Availability

Supplementary data are available in supplementary files, All plastomes are submitted to GenBank (https://www.ncbi.nlm.nih.gov/nucleotide/) with their accession numbers given in Table S1.

## Abbreviations

*aa*: Amino acid
*BI*: Bayesian inference
*BS*: Bootstrap
*CDS*: Coding sequence
*cpDNA*: Chloroplast DNA
*IGS*: Inter-genic spacer
*IR*: Inverted repeat
*ITS*: Internal transcribed spacer
*LRT*: Likelihood ratio test
*LSC*: Large single copy region
*ML*: Maximum likelihood
*PEP*: Plastid-encoded polymerase
*PP*: Posterior probability
*SSC*: Small single copy region
