# Supplementary material for: Insights into adaptive evolution of plastomes in Stipa L. (Poaceae)
Source: BMC Plant Biol. 2022 Nov 14;22:525. doi: 10.1186/s12870-022-03923-z (PMC9661759; doi:10.1186/s12870-022-03923-z)

**Fig. S1. Distribution of substitution rates across eleven genes tested towards selective pressure.**

Calculated in HYPhy using the model of evolution according to the results of previous model testing.

*ccsA*
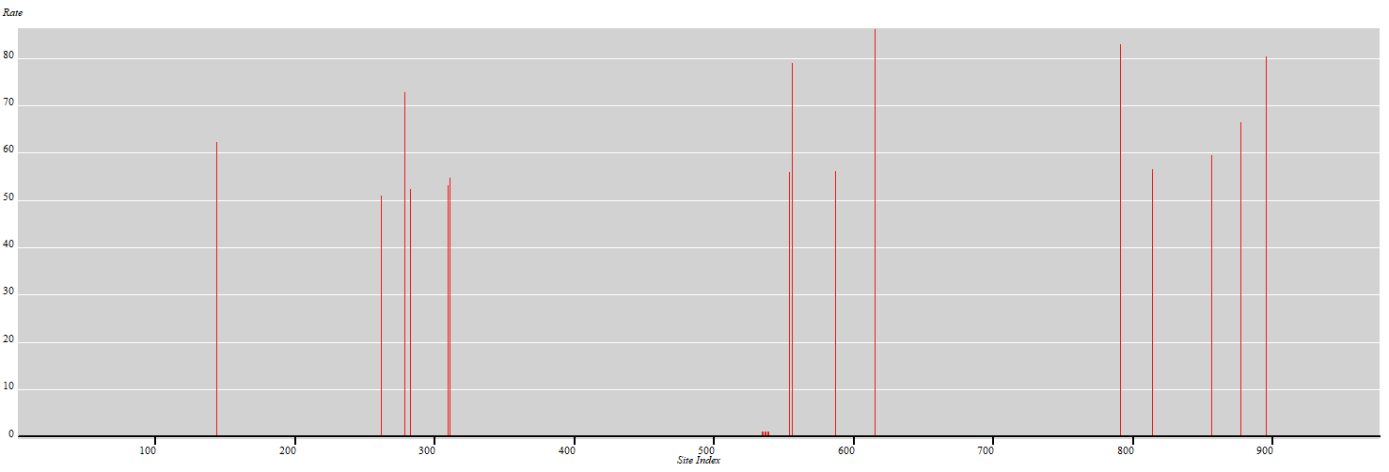


*matK*
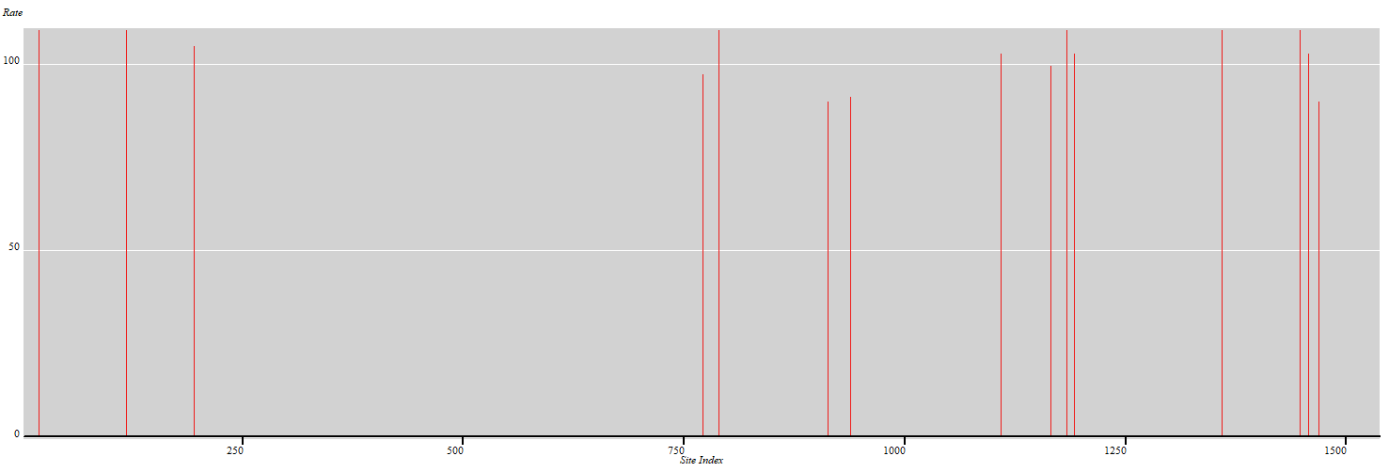


*ndhC*

*
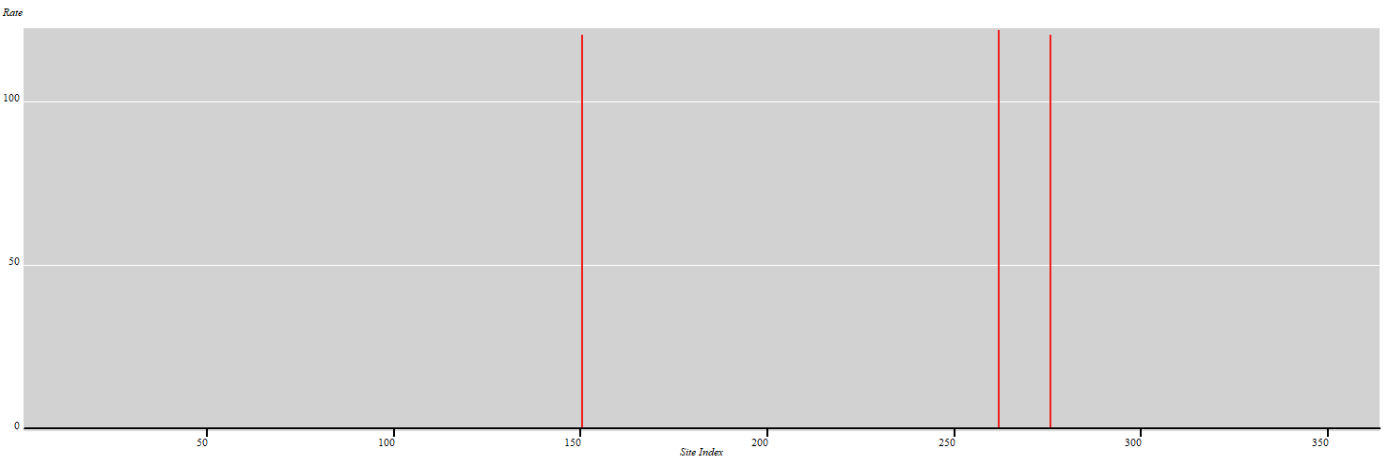
*

*ndhF*
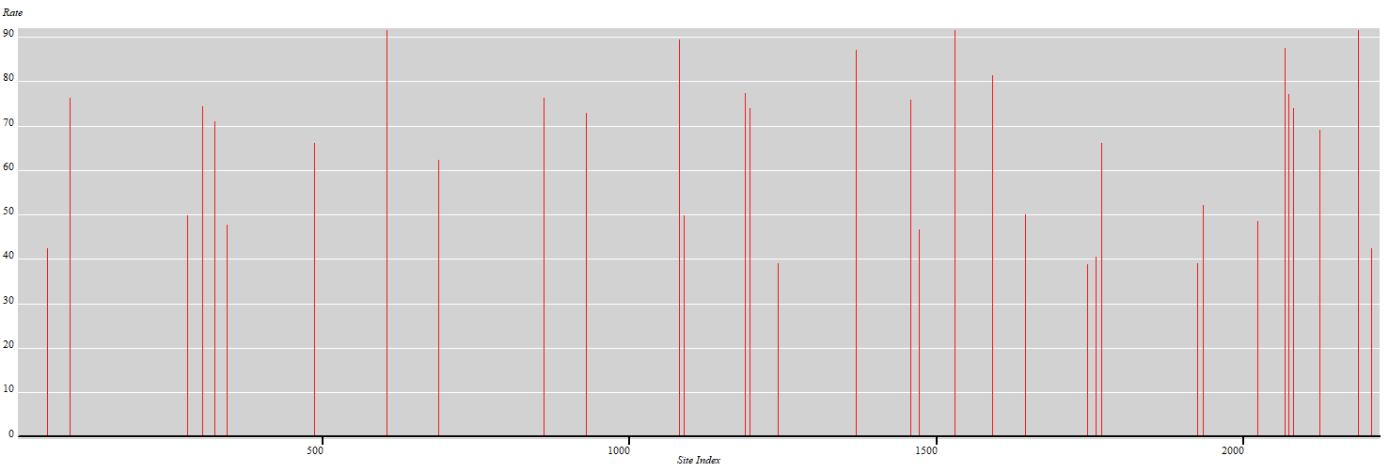


*ndhK*
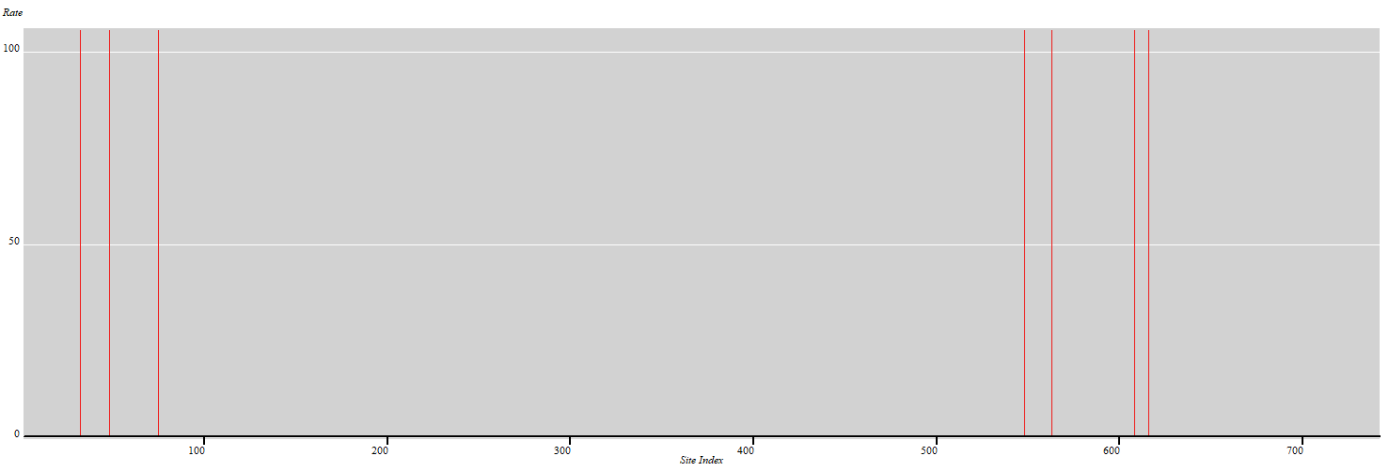


*rbcL*
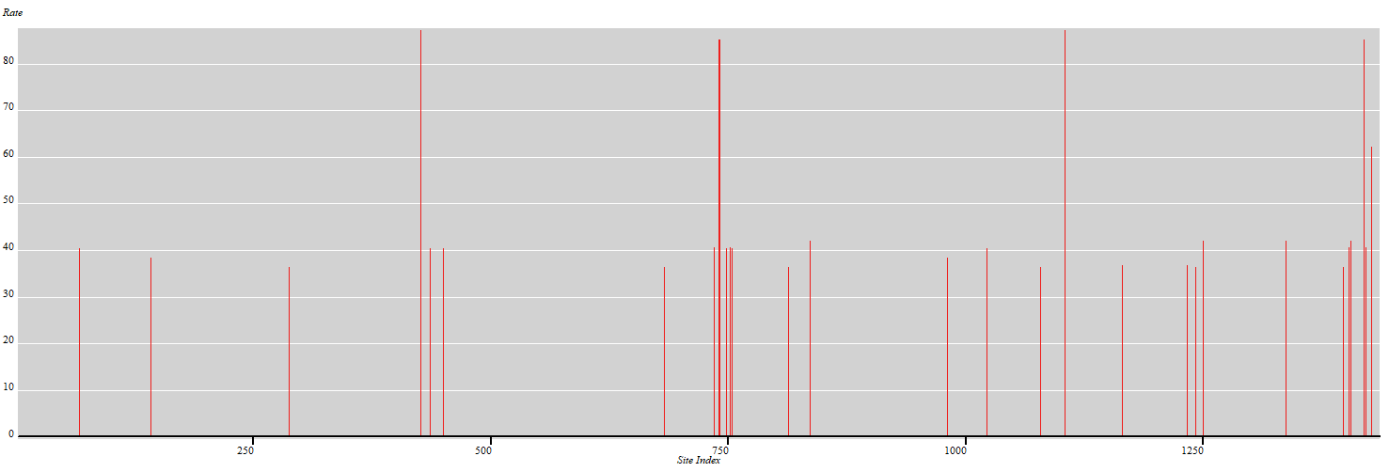


*rpoA*

*
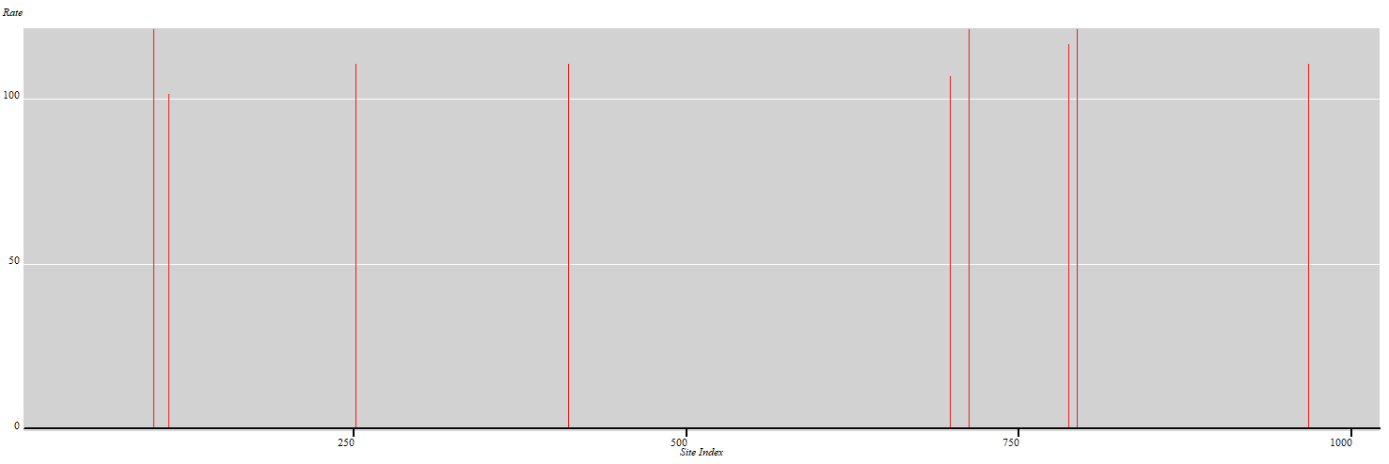
*

*rpoC1*
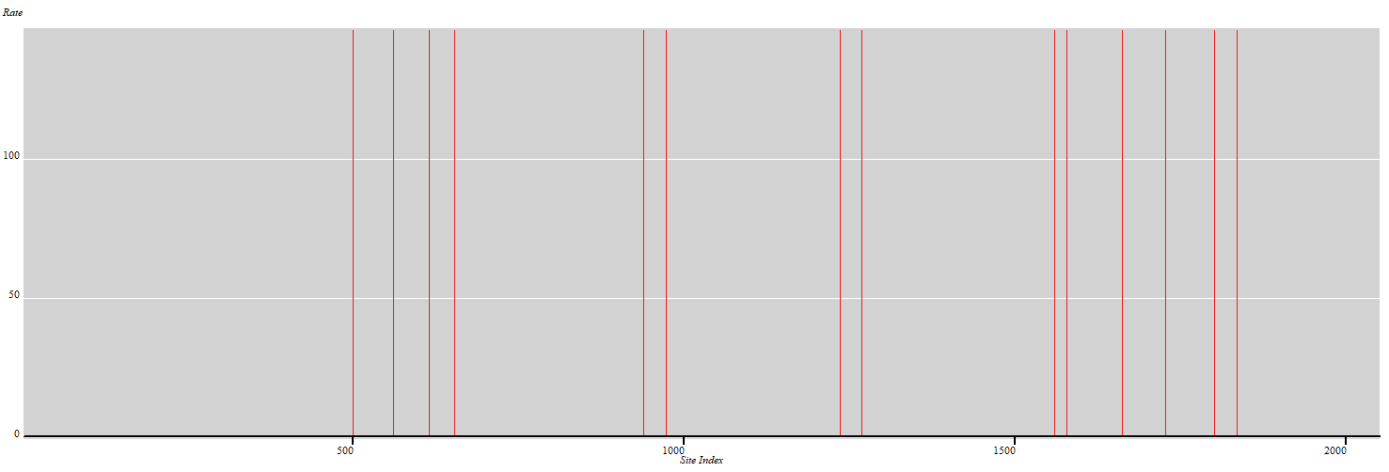


*rpoC2*
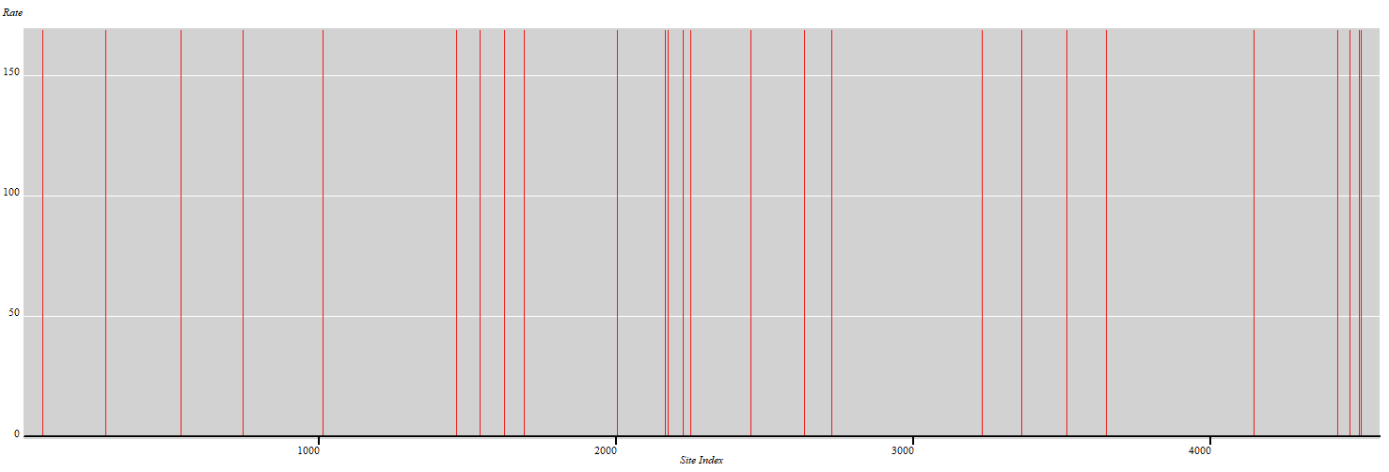


*rps8*

*
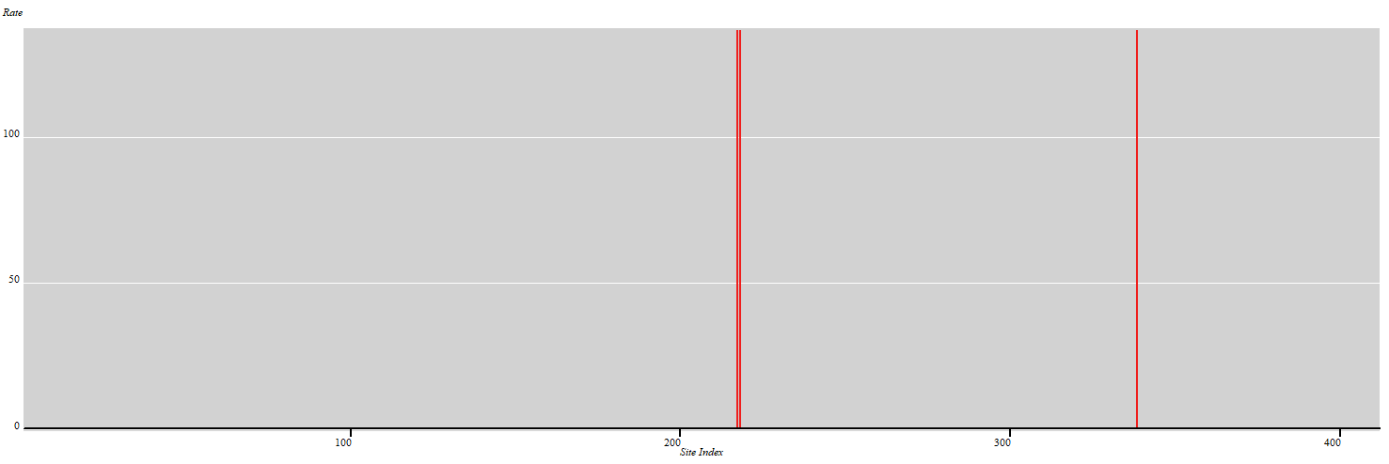
*

*rps11*
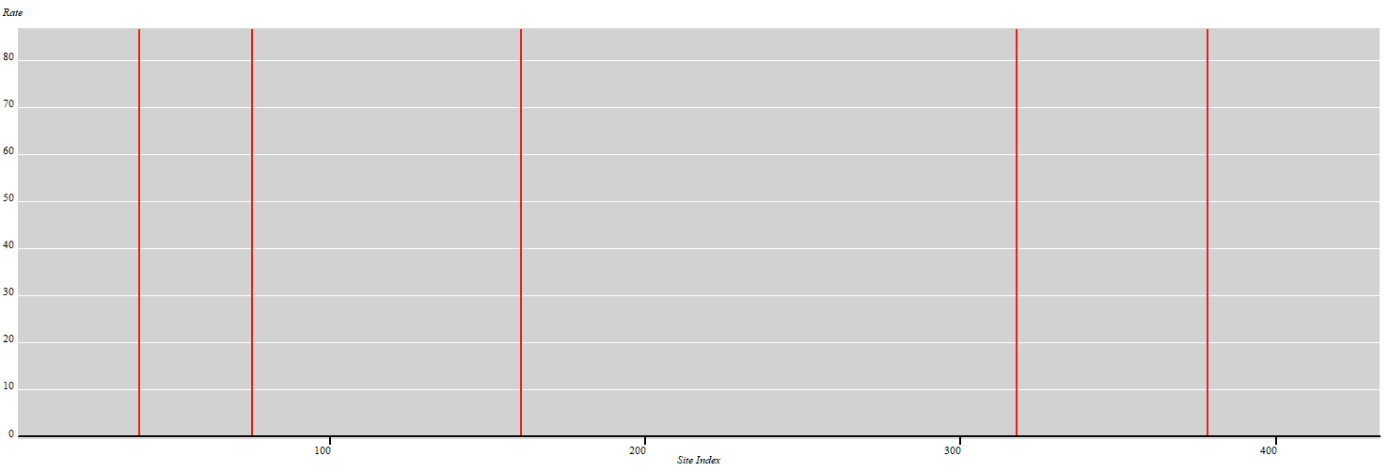

Supplement: Supplementary file 4 — Additional file 4: Figure S1. Distribution of substitution rates across 11 genes as calculated in HyPhy using the GTR model of evolution. [file 12870_2022_3923_MOESM4_ESM.docx]
