# Supplementary material for: Insights into adaptive evolution of plastomes in Stipa L. (Poaceae)
Source: BMC Plant Biol. 2022 Nov 14;22:525. doi: 10.1186/s12870-022-03923-z (PMC9661759; doi:10.1186/s12870-022-03923-z)
Supplement: Supplementary file 5 — Additional file 5: Figure S2. The likelihood of adaptive pressure occurrence in particular CDS. The plots show the results of the Z-test, carried out for the 15-codon-long fragments. The values shown in the Y-axis (Z-scores) above 2.33 (blue horizontal line) correspond to the reliability level of 99%. Values above 3.09 (red horizontal line) correspond to the reliability of 99.9%. [file 12870_2022_3923_MOESM5_ESM.docx]

**Fig. S2. The likelihood of adaptive pressure occurrence in particular CDS.**
